# Deficiency of angiotensin-converting enzyme 2 causes deterioration of cognitive function

**DOI:** 10.1038/npjamd.2016.24

**Published:** 2016-10-20

**Authors:** Xiao-Li Wang, Jun Iwanami, Li-Juan Min, Kana Tsukuda, Hirotomo Nakaoka, Hui-Yu Bai, Bao-Shuai Shan, Harumi Kan-no, Masayoshi Kukida, Toshiyuki Chisaka, Toshifumi Yamauchi, Akinori Higaki, Masaki Mogi, Masatsugu Horiuchi

**Affiliations:** 1 Department of Molecular Cardiovascular Biology and Pharmacology, Ehime University, Graduate School of Medicine, Ehime, Japan; 2 Department of Cardiology, Pulmonology, Hypertension and Nephrology, Ehime University, Graduate School of Medicine, Ehime, Japan; 3 Department of Pediatrics, Ehime University, Graduate School of Medicine, Ehime, Japan

## Abstract

The classical renin–angiotensin system (RAS), known as the angiotensin (Ang)-converting enzyme (ACE)/Ang II/Ang II type 1 (AT1) receptor axis, induces various organ damages including cognitive decline. On the other hand, the ACE2/Ang-(1–7)/Mas receptor axis has been highlighted as exerting antagonistic actions against the classical RAS axis in the cardiovascular system. However, the roles of the ACE2/Ang-(1–7)/Mas axis in cognitive function largely remain to be elucidated, and we therefore examined possible roles of ACE2 in cognitive function. Male, 10-week-old C57BL6 (wild type, WT) mice and ACE2 knockout (KO) mice were subjected to the Morris water maze task and Y maze test to evaluate cognitive function. ACE2KO mice exhibited significant impairment of cognitive function, compared with that in WT mice. Superoxide anion production increased in ACE2KO mice, with increased mRNA levels of NADPH oxidase subunit, p22^phox^, p40^phox^, p67^phox^, and gp91^phox^ in the hippocampus of ACE2KO mice compared with WT mice. The protein level of SOD3 decreased in ACE2KO mice compared with WT mice. The AT1 receptor mRNA level in the hippocampus was higher in ACE2KO mice compared with WT mice. In contrast, the AT2 receptor mRNA level in the hippocampus did not differ between the two strains. Mas receptor mRNA was highly expressed in the hippocampus compared with the cortex. Brain-derived neurotrophic factor (BDNF) mRNA and protein levels were lower in the hippocampus in ACE2KO mice compared with WT mice. Taken together, ACE2 deficiency resulted in impaired cognitive function, probably at least in part because of enhanced oxidative stress and a decrease in BDNF.

## Introduction

The renin–angiotensin system (RAS) has important roles in blood pressure regulation and cardiovascular function. Recently, roles of RAS in cognitive function have been highlighted. Li *et al.*^1^ reported that angiotensin (Ang) II type 1 (AT1) receptor blockers (ARBs) were associated with significant reduction in the incidence and progression of Alzheimer's disease and dementia compared with Ang-converting enzyme (ACE) inhibitors and other cardiovascular drugs in a population of 819,491 predominantly male participants aged 65 years or more with cardiovascular disease. Chiu *et al.*^2^ reported that ARBs may be associated with a reduced risk of dementia in high-vascular-risk individuals, and that patients exposed to ARBs at higher cumulative doses experienced greater protection from dementia and its subtypes, based on a population-based cohort study of 24,531 matched pairs with data from the Taiwan National Health Insurance Research Database.

These clinical results support the idea that both AT1 receptor blockade and Ang II type 2 (AT2) receptor stimulation by unbound Ang II are important in the neuroprotective effect of ARBs.^3^ In addition to the AT2 receptor, recent experimental studies have also demonstrated the existence of novel pathways beyond the classical actions of RAS. A new axis of RAS, the ACE2/Ang-(1–7)/Mas axis has been highlighted as the counteracting partner of the ACE/Ang II/AT1 receptor.^4^ Ang-(1–7) is finally produced from Ang I or Ang II by the catalytic activity of ACE2, and the discovery that Ang-(1–7) opposes the pressor, proliferative, fibrotic, and thrombotic actions mediated by Ang II via the AT1 receptor has contributed to the realization that RAS is composed of two opposing arms.^5,6^

Regenhardt *et al.*^7^ reported the cerebroprotective actions of ACE2/Ang-(1–7) in ischemic stroke and hemorrhagic stroke via anti-inflammatory effects within the brain parenchyma. Moreover, the ACE2/Ang-(1–7)/Mas axis in the brain was also reported to have an important role in the regulation of blood pressure centrally and has an inhibitory neuromodulatory role in hypothalamic noradrenergic neurotransmission, and also acts as a cerebroprotective component by reducing neuronal apoptosis.^8^ Xie *et al.*^9^ recently reported that Ang-(1–7) significantly alleviated chronic cerebral hypoperfusion-induced cognitive deficits in rats. However, in spite of these previous reports, the roles of the ACE2/Ang-(1–7)/Mas axis in cognitive function are largely unknown and remain to be elucidated in more detail. In this study, we focused on the effects of deficiency of ACE2 on cognitive function, employing ACE2-deficient mice.

## Results

### Body weight, brain weight, and blood pressure

There were no significant differences in body weight, brain weight, and brain weight/body weight ratio and blood pressure between 10-week-old ACE2KO mice and wild-type (WT) mice (Supplementary Table 1). Heart rate in ACE2KO mice increased significantly compared with that in WT mice (Supplementary Table 1).

### ACE2-deficient mice showed impaired cognitive function

**Figure 1:**
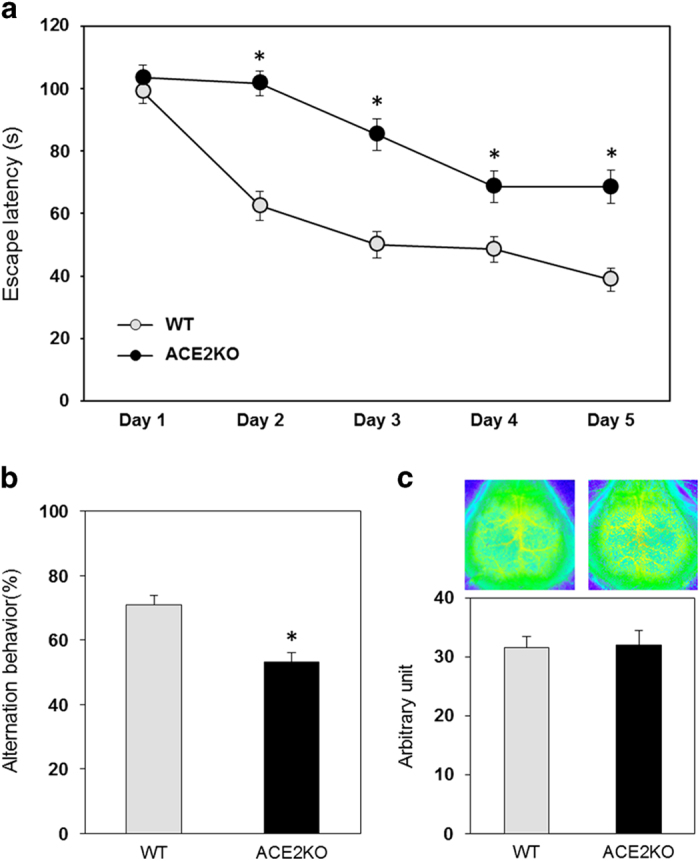
Effect of ACE2 deficiency on cognitive function and cerebral blood flow. (**a**) Cognitive function was assessed using the Morris water maze as described in Materials and methods. Data are expressed as mean±s.e.m. (*n*=18–23 for each group). *P<0.05 versus WT mice. (**b**) Cognitive function was assessed using Y maze test as described in Materials and methods. Data are expressed as mean±s.e.m. (*n*=11–12 for each group). *P<0.05 versus WT mice. (**c**) Cerebral blood flow was measured with a laser speckle blood flow imager as described in Materials and methods. Representative images of each group are shown in the upper panels, and quantitative analysis is shown in the lower panel. The value in each mouse was determined as the average of 10 image pixels. Data are expressed as mean±s.e.m. (*n*=9–10 for each group). ACE2, angiotensin-converting enzyme 2; WT, wild type.

**Figure 2:**
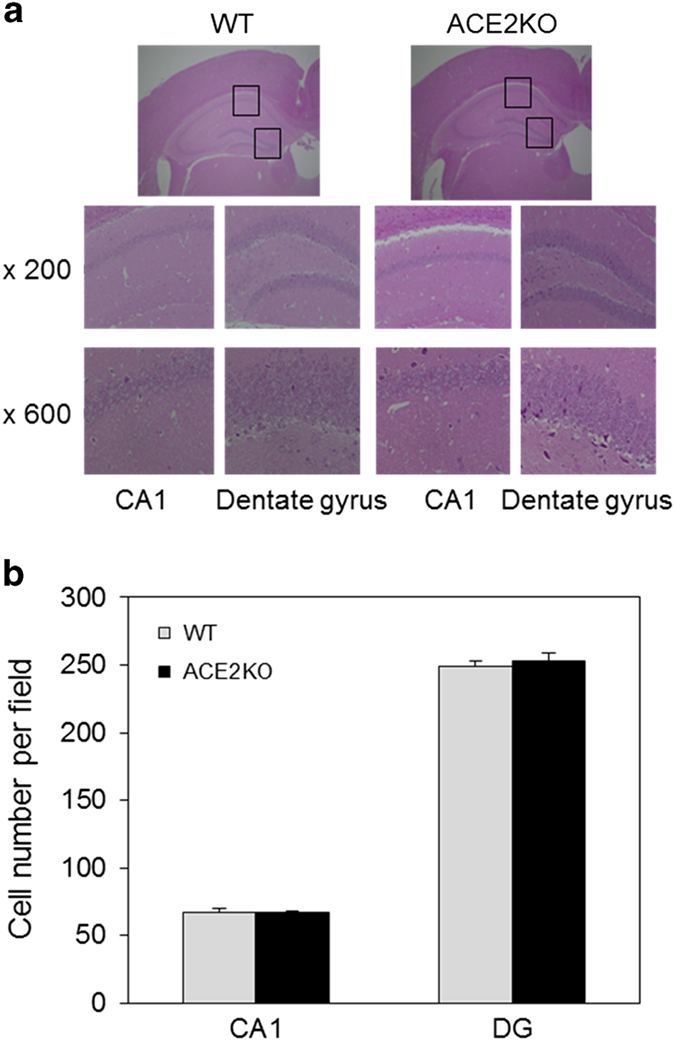
Effect of ACE2 deficiency on morphology and cell number in the hippocampus. (**a**) The hippocampus was examined morphologically by HE staining as described in Materials and methods. Representative photos are shown in the upper panel (×40), and the central area of CA1 and the dentate gyrus are shown in the middle panel (×200) and lower panel (×600). (**b**) Cell numbers were counted in the central area of CA1 and dentate gyrus under ×600 magnification. The value in each mouse was determined from the average of four different slices (*n*=2). ACE2, angiotensin-converting enzyme 2; HE, hematoxylin–eosin.

Spatial learning was evaluated by the Morris water maze test. ACE2KO mice exhibited shorter swim latency compared with that in WT mice from day 2 to day 5 (Figure 1a). Moreover, in the Y maze test, we observed that ACE2KO mice showed cognitive decline compared with WT mice (Figure 1b), suggesting that ACE2 deficiency caused impaired cognitive function. However, ACE2KO mice did not show a significant difference in cerebral blood flow (CBF) compared with WT mice after the Morris water maze test (Figure 1c). We next examined possible morphological changes in the hippocampus of ACE2KO mice. Hematoxylin–eosin staining did not show any significant structural differences in the hippocampus between ACE2KO and WT mice (Figure 2a). Moreover, we observed that the cell numbers calculated in the central area of CA1 and dentate gyrus of the hippocampus did not differ between the two strains of mice (Figure 2b).

### Expression of AT1 receptor, AT2 receptor, and Mas receptor mRNA in the hippocampus of ACE2-deficient mice

**Figure 3:**
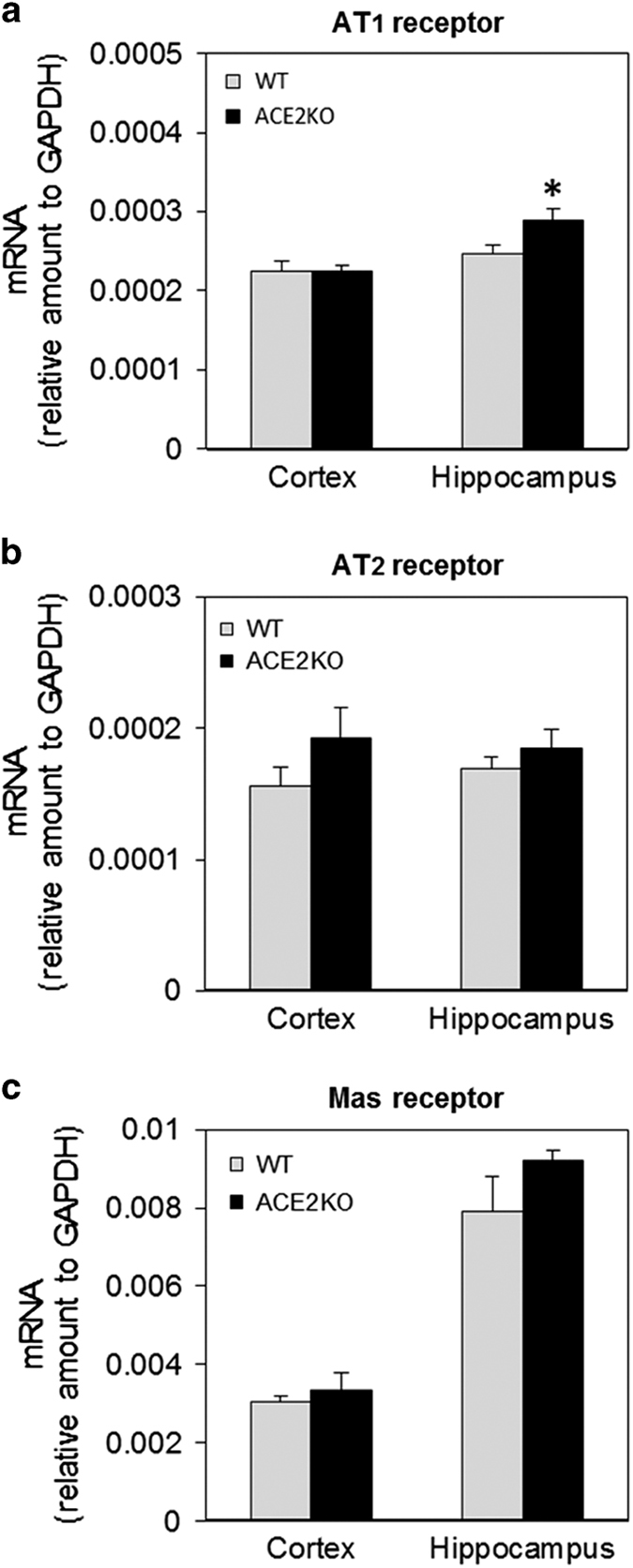
Expression of AT1 receptor, AT2 receptor, and Mas receptor mRNA. AT1 (**a**), AT2 (**b**), and Mas (**c**) receptor mRNA were determined using real-time RT-PCR as described in Materials and methods. Values are expressed as mean±s.e.m. (*n*=7–8 for each group). *P<0.05 versus WT mice. AT1 receptor, angiotensin II type 1 receptor; AT2 receptor, angiotensin II type 2 receptor; RT-PCR, reverse transcriptase polymerase chain reaction; WT, wild type.

We next examined the mRNA levels of the AT1, AT2, and Mas receptors (Figure 3a–c). AT1 receptor expression in the hippocampus was higher in ACE2KO mice compared with that in WT mice, with no significant difference in the cortex (Figure 3a). AT2 receptor expression both in the cortex and hippocampus did not differ between the two strains of mice (Figure 3b). Mas receptor mRNA was highly expressed in the hippocampus compared with that in the cortex, with no significant difference between ACE2KO and WT mice (Figure 3c).

### Enhanced oxidative stress in the hippocampus of ACE2-deficient mice

**Figure 4:**
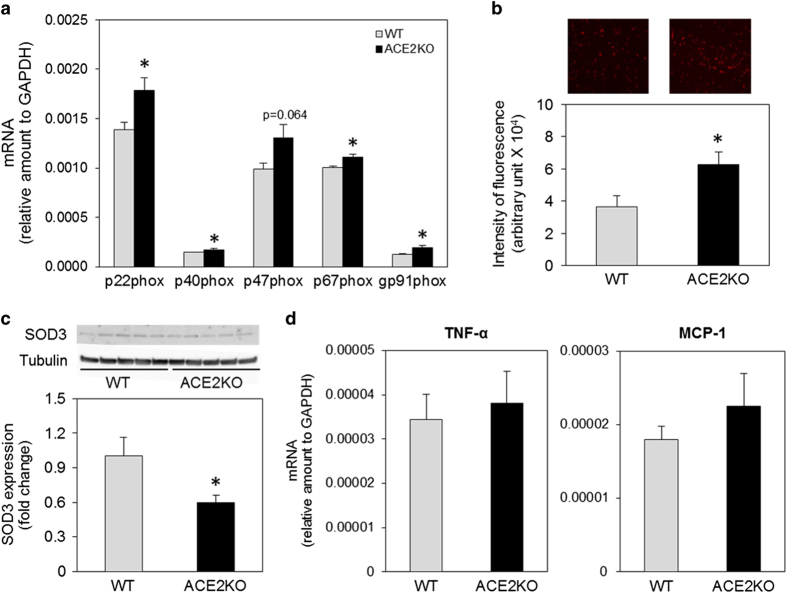
Effect of ACE2 deficiency on oxidative stress and inflammation. (**a**) NADPH oxidase p22^phox^, p40^phox^, p47^phox^, P67^phox^, and gp91^phox^ subunit mRNA was detected using real-time RT-PCR as described in Materials and methods. Data are expressed as mean±s.e.m. (*n*=8 for each group). *P<0.05 versus WT mice. (**b**) Superoxide anion was detected using dihydroethidium as described in Materials and methods. Representative photos are shown in the upper panel, and the lower panel shows quantitative analysis. The value in each mouse was determined from the average of eight areas of the hippocampus. Data are expressed as mean±s.e.m. (*n*=18–20 for each group). (**c**) The protein level of SOD3 was determined by western blotting as described in Materials and methods. Representative blots are shown in the upper panel, and the lower panel shows quantitative analysis. Data are expressed as mean±s.e.m. (*n*=9). (**d**) TNF-α and MCP-1 mRNAs were detected using real-time RT-PCR as described in Materials and methods. Data are expressed as mean±s.e.m. (*n*=8 for each group). ACE2, angiotensin-converting enzyme 2; MCP-1, monocyte chemoattractant protein-1; NADPH, nicotinamide adenosine dinucleotide; RT-PCR, reverse transcriptase polymerase chain reaction; SOD3, superoxide dismutase 3; TNF-α, tumor necrosis factor α; WT, wild type.

NADPH oxidase subunit p22^phox^, p40^phox^, p67^phox^, and gp91^phox^ mRNA levels in the hippocampus of ACE2KO mice showed a significant increase compared with those in WT mice (Figure 4a). We next examined superoxide anion production in the hippocampus by dihydroethidium staining and observed that superoxide anion production in ACE2KO mice significantly increased compared with that in WT mice (Figure 4b). The protein level of SOD3 decreased in ACE2KO mice compared WT mice (Figure 4c). We also examined mRNA levels of inflammatory cytokines, such as tumor necrosis factor-α and monocyte chemoattractant protein-1, in the hippocampus and observed no significant difference between ACE2KO and WT mice (Figure 4d).

### Expression of brain-derived neurotrophic factor in the hippocampus of ACE2-deficient mice

**Figure 5:**
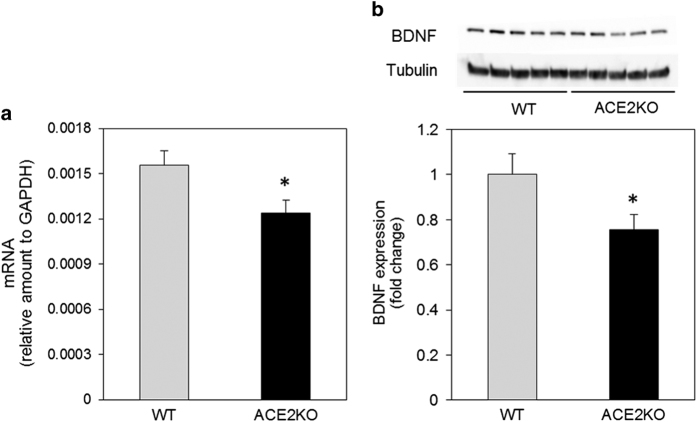
Effect of ACE2 deficiency on BDNF expression. (**a**) BDNF mRNA was detected using real-time RT-PCR as described in Materials and methods. Data are expressed as mean±s.e.m. (*n*=8 for each group). *P<0.05 versus WT mice. (**b**) BDNF protein was detected by western blot as described in Materials and methods. Values are expressed as mean±s.e.m. (*n*=14 for each group). ACE2, angiotensin-converting enzyme 2; BDNF, brain-derived neurotrophic factor; RT-PCR, reverse transcriptase polymerase chain reaction; WT, wild type.

Brain-derived neurotrophic factor (BDNF) is a member of the neurotrophin family and ARB is known to increase its expression in the brain.^10^ Therefore, we examined the expression of BDNF in the hippocampus. We observed a lower BDNF mRNA level in the hippocampus in ACE2KO mice compared with WT mice (Figure 5a). The protein level of BDNF determined by western blot decreased in the hippocampus in ACE2KO mice compared with that in WT mice (Figure 5b).

### Effect of Ang-(1–7) and telmisartan on ACE2-deficient mice

**Figure 6:**
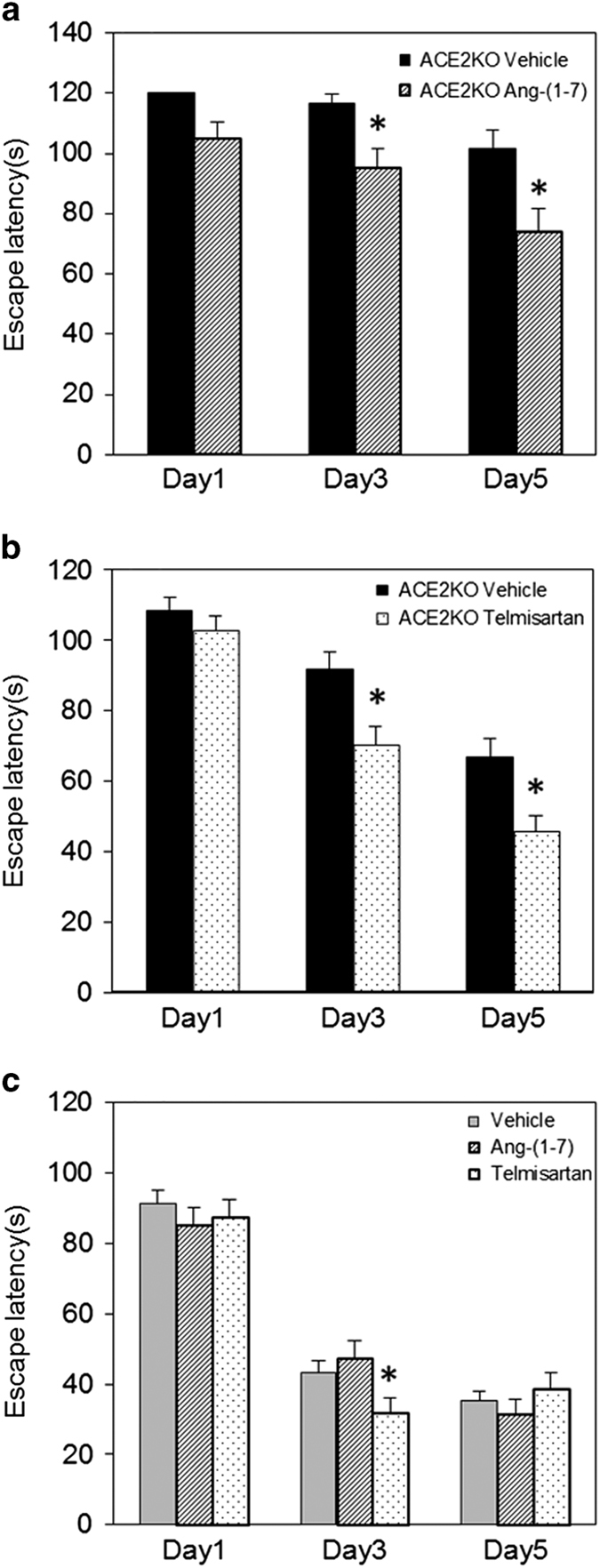
Effect of Ang-(1–7) and telmisartan on ACE2-deficient mice. (**a**) Cognitive function was analyzed using Morris water maze test as described in Materials and methods. Ang-(1–7) was administered intraperitoneally using osmotic minipump at the concentration of 0.5 mg/kg/day. Data are expressed as mean±s.e.m. (*n*=5–8 for each group). *P<0.05 versus vehicle group. (**b**) Cognitive function was analyzed using Morris water maze test as described in Materials and methods. Telmisartan was administered in drinking water at the concentration of 1.0 mg/kg/day. Values are expressed as mean±s.e.m. (*n*=16 for each group). *P<0.05 versus vehicle group. (**c**) Effect of Ang-(1–7) and telmisartan on cognitive function in WT mice. Data are expressed as mean±s.e.m. (*n*=15–30 for each group). *P<0.05 versus vehicle group. ACE2, angiotensin-converting enzyme 2; WT, wild type.

**Figure 7:**
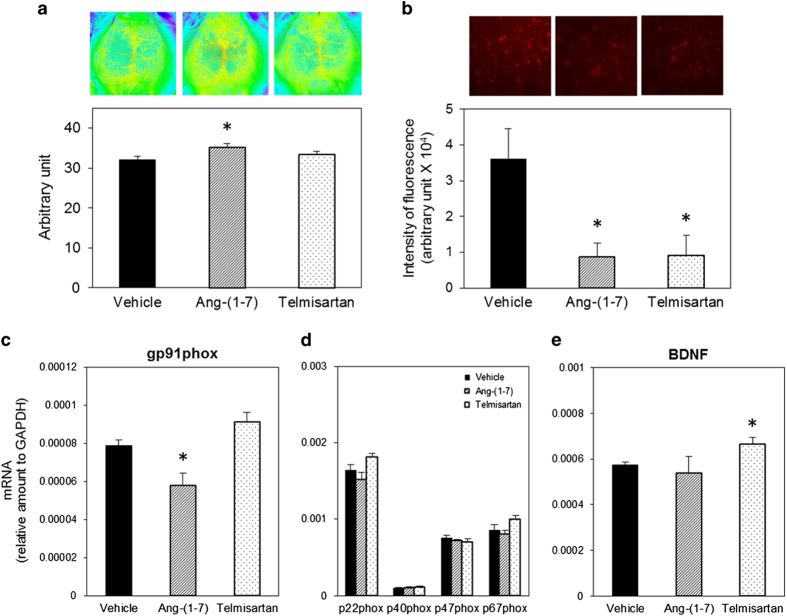
Mechanisms of improved cognitive function by Ang-(1–7) and telmisartan in ACE2-deficient mice. (**a**) Cerebral blood flow was measured with a laser speckle blood flow imager as described in Materials and methods. Representative images of each group are shown in the upper panels, and quantitative analysis is shown in the lower panel. The value in each mouse was determined as the average of 10 image pixels. Data are expressed as mean±s.e.m. (*n*=6–9 for each group). *P<0.05 versus vehicle group. (**b**) Superoxide anion was detected using dihydroethidium as described in Materials and methods. Representative photos are shown in the upper panel, and the lower panel shows quantitative analysis. The value in each mouse was determined from the average of eight areas of the hippocampus. Data are expressed as mean±s.e.m. (*n*=4–6 for each group). *P<0.05 versus vehicle group. (**c** and **d**) NADPH oxidase gp91phox p22phox, p40phox, p47phox, and P67phox subunit mRNAs were measured using RT-PCR as described in Materials and methods. Data are expressed as mean±s.e.m. (*n*=7 for each group). *P<0.05 versus vehicle group. (**e**) BDNF mRNA was measured using real-time RT-PCR as described in Materials and methods. Data are expressed as mean±s.e.m. (*n*=7–10 for each group). *P<0.05 versus vehicle group. ACE2, angiotensin-converting enzyme 2; BDNF, brain-derived neurotrophic factor; RT-PCR, reverse transcriptase polymerase chain reaction.

Administration of Ang-(1–7) intraperitoneally using osmotic minipump at the concentration of 0.5 mg/kg/day attenuated the cognitive decline in ACE2KO mice (Figure 6a). Administration of telmisartan improved impaired cognitive function in ACE2KO mice (Figure 6b). In contrast, treatment with Ang-(1–7) or telmisartan did not influence on cognitive function in WT mice on day 5 (Figure 6c). We measured CBF and superoxide anion production to find possible mechanisms in which Ang-(1–7) and telmisartan improved impairment of cognitive function in ACE2KO mice. Ang-(1–7) increased CBF, but telmisartan did not (Figure 7a). In contrast, superoxide anion production was significantly decreased by treatment with Ang-(1–7) or telmisartan, respectively (Figure 7b). Interestingly, Ang-(1–7) decreased only mRNA expression of gp91phox, but telmisartan did not influence any mRNA expressions (Figures 7c and d). On the other hand, telmisartan significantly increased the mRNA level of BDNF, but Ang-(1–7) did not (Figure 7e).

### Effect of antioxidant tempol on cognitive function in ACE2-deficient mice

**Figure 8:**
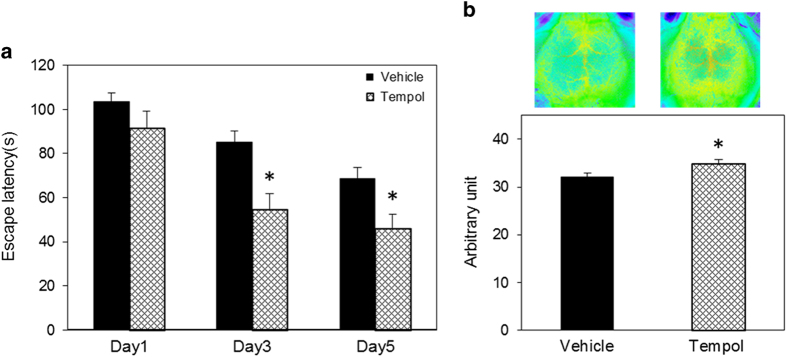
Effect of antioxidant tempol on cognitive function in ACE2-deficient mice. (**a**) Cognitive function was evaluated using Morris water maze test as described in Materials and methods. Tempol was injected intraperitoneally once a day for 2 weeks at the concentration of 100 mg/kg/day. Data are expressed as mean±s.e.m. (*n*=7–18 for each group). *P<0.05 versus vehicle group. (**b**) Cerebral blood flow was measured with a laser speckle blood flow imager as described in Materials and methods. Representative images of each group are shown in the upper panels, and quantitative analysis is shown in the lower panel. The value in each mouse was determined as the average of 10 image pixels. Data are expressed as mean±s.e.m. (*n*=7–9 for each group). *P<0.05 versus vehicle group. ACE2, angiotensin-converting enzyme 2.

We administered superoxide dismutase mimetic, tempol, to ACE2KO mice to directly check the effect of oxidative stress on cognitive function. Tempol remarkably improved the impairment of cognitive function in ACE2KO mice (Figure 8a). Interestingly, CBF significantly increased in the tempol-treated group than in the vehicle group (Figure 8b).

## Discussion

The Ang-(1–7)/ACE2/Mas receptor axis has been highlighted as the counterpart of the Ang II/ACE/AT1 receptor axis, and Ang-(1–7) in the brain is known to not only exert effects related to blood pressure regulation as the depressor arm, but also to act as a cerebroprotective component of RAS by reducing cerebral infarct size and neuronal apoptosis.^8^ ACE2 is widely expressed in brain areas involved in both the central regulation of blood pressure and cardiovascular function and disease, and the ACE2 level appears to be strongly regulated by other components of RAS.^11^ Immunostaining for Ang-(1–7) has been observed in the areas of the brain, including the supra-optic and paraventricular nucleus (PVN) of the hypothalamus, neurons from the hypothalamus, and brainstem.^8^ Furthermore, the level of Ang-(1–7) was detected from the hippocampus using high-performance liquid chromatography assay and increased during the acute and silent phases of pilocarpine-induced epilepsy.^12^ Pereira *et al.*^13^ show that Ang-(1–7) is the main metabolite of Ang I in rat hippocampi. These results also suggest that the ACE2/Ang-(1–7)/Mas receptor axis in the brain is essential for cognitive function. Concentration of Ang-(1–7) in the hippocampus of ACE2KO mice tended to decrease, without significant difference compared with that of WT mice, and concentration of Ang II in the hippocampus did not differ between ACE2KO and WT mice (data not shown). The Mas receptor is abundant in the hippocampus, amygdala, anterodorsal thalamic nucleus, cortex, and hypoglossal nucleus in the rat brain, and is predominantly present in neurons.^14^ AT1 receptor immunoreactivity was reported to be located in neuron of the hippocampus in rats.^15,16^ It has been reported that strong AT2 receptor immunoreactivity and protein were distributed in the hippocampal neuron of human.^17^ It has been reported that strongest Mas protein expression in the mouse brain was detected in the dentate gyrus of the hippocampus and within the piriform cortex; however, Mas protein expression is not restricted to these areas, as Mas-immunopositive neurons were also seen in different parts of the cortex, hippocampus, amygdala, basal ganglia, thalamus, and hypothalamus, suggesting that Ang-(1–7) signaling may have a role in synaptic plasticity, learning, memory, and emotion.^18^ Lazaroni *et al.*^19^ demonstrated that Mas ablation, as well as blockade of Mas in the CA1–hippocampus, impaired object recognition memory (ORM), and that blockade of the AT1, but not AT2, Ang II receptor reversed ORM impairment in Mas-deficient mice. Consistent with these observations, we demonstrated a higher level of Mas receptor mRNA in the hippocampus compared with that in the cortex, with no significant difference between WT and ACE2KO mice. ACE2 expression and AT1 receptor levels seem to be intimately connected.^8^ AT1 receptor activation by Ang II increased the expression and activity of NADPH oxidase in the rat brain,^20^ and blockade of AT1 receptor signaling by losartan ameliorated renal NADPH oxidase expression in hypertensive model induced by nitric oxide (NO) inhibition.^21^ We also demonstrated that the AT1 receptor mRNA level was higher in the ACE2KO mouse hippocampus. These results suggest the possibility that enhanced AT1 receptor stimulation in the hippocampus of ACE2KO may contribute to an increase in oxidative stress, especially in the hippocampus.

Oxidative stress has been reported to have a key role in cognitive impairment. Jiang *et al*.^22^ reported that intracerebroventricular infusion of Ang-(1–7) for 4 weeks significantly reduced inducible NO synthase and the NADPH oxidase subunit gp91 in the brain of SHR, and that these antioxidative and anti-apoptotic effects caused by chronic infusion of Ang-(1–7) in spontaneously hypertensive rat (SHR) were accompanied by a reduction in the expression of Ang II and AT1 receptor, and were independent of blood pressure reduction. NADPH oxidase is the major source of superoxide anion; however, the mechanism of NADPH oxidase activation by Ang-(1–7) has not been elucidated. We also observed in this study that mRNA levels of NADPH oxidase subunits p22^phox^, p40^phox^, p67^phox^, and gp91^phox^, and superoxide anion production in the hippocampus of ACE2KO mice was higher compared with that in WT mice. Moreover, expression of SOD3 in the hippocampus decreased in ACE2KO mice. Previous studies demonstrated that Ang-(1–7) increased the bradykinin level, NO release, and endothelial NO synthase expression in the brain, suggesting a further mechanism of Ang-(1–7) against cerebrovascular disease.^23^ Central administration of Ang-(1–7) stimulated NO release and upregulated endothelial NO synthase expression following focal cerebral ischemia/reperfusion in rats.^24–28^ The counterbalance between the Ang-(1–7)-NO and Ang II/AT1 receptor superoxide anion signaling pathways is important in neurocardiovascular responses.^29^ We have not examined the NO level in ACE2KO mice; however, we can expect that imbalance of reactive oxygen species and NO may contribute to the impaired cognitive function in ACE2KO mice. Treatment with antioxidant, tempol, improved cognitive decline in ACE2KO mice, suggesting that enhanced oxidase stress impairs cognitive function in ACE2KO mice.

BDNF is a member of the protein family of mammalian neurotrophins. The main function of BDNF is to enhance synaptic transmission, facilitate synaptic plasticity, promote synaptic growth, and enhance cognition in the adult brain.^30^ BDNF has emerged as a major regulator of activity-dependent plasticity at excitatory synapses in the mammalian central nervous system, and the role of this neurotrophin in the regulation of hippocampal long-term potentiation (LTP) has been focused on in terms of underlying learning and memory processes.^31^ BDNF has also been reported to decrease in the temporal cortex in the brain of Alzheimer patients, independent of BDNF polymorphisms.^23^ The level of expression and secretion of BDNF were reported to be associated with neurodegenerative disorders, such as Alzheimer's disease and Huntington’s disease, as well as psychiatric disorders, including depression and substance abuse.^32^ In this study, we observed that BDNF mRNA and protein levels were lower in ACE2KO mice compared with that in WT mice. Previous reports suggest that peroxisome proliferator-activated receptor γ (PPARγ) expression decreases in the heart of ACE2KO^33^ and central activation of PPARγ improves BDNF expression.^34^ Therefore, PPARγ may be involved in such a reduced BDNF expression in ACE2KO mice. Furthermore, treatment with Ang-(1–7) or telmisartan improved the impairment of cognitive function in ACE2KO mice because of different pathways. Ang-(1–7) decreased superoxide anion production and gp91phox mRNA expression, whereas telmisartan decreased superoxide anion production and increased BDNF expression.

Hellner *et al.*^35^ reported that Ang-(1–7) enhanced LTP in the CA1 region of the hippocampus, and that genetic deletion of the Mas receptor abolishes Ang-(1–7)-induced enhancement of LTP. Ang-(1–7) has also been reported to exert a protective role against blood–brain barrier damage through the balance of tissue inhibitor of metalloproteinase 1 and matrix metalloproteinase-9 hemopexin domain.^36^ Moreover, Liu *et al.*^37^ found that Aβ43 was able to be converted into Aβ42 and Aβ40 in mouse brain lysates, and identified the brain Aβ43-to-Aβ42-converting enzyme to be ACE2. They also reported that ACE2 tended to decrease in the serum of Alzheimer's disease patients compared with normal controls. These results led us to speculate that other possible mechanisms might be involved in impaired cognitive function in ACE2KO mice. These possible mechanisms remain to be investigated.

In conclusion, this study showed that ACE2 deficiency in the mouse resulted in impaired cognitive function, and we propose that the mechanisms of the cognitive decline may be associated at least in part with increased oxidative stress as well as a decreased level of BDNF in the hippocampus in ACE2KO mice. Our study could contribute to further understanding of the roles of the ACE2/Ang-(1–7)/Mas axis in cognitive function, and encourage us to examine the more-detailed mechanisms of this protective arm of RAS in preventing cognitive decline, especially in lifestyle-related diseases such as hypertension and diabetes.

## Materials and methods

This study was performed in accordance with the National Institutes of Health guidelines for the use of experimental animals. All animal studies were reviewed and approved by the Animal Studies Committee of Ehime University.

### Animals

Adult male C57BL6 mice (Clea Japan, Tokyo, Japan) and ACE2 knockout (KO) mice were used (C57BL/6J background, provided by Otsuka Pharmaceutical, Tokyo, Japan).^38^ Mice were kept in a room in which lighting was controlled (12 h on, 12 h off) and temperature was kept at 25 °C. They were given a standard diet (MF diet, Oriental Yeast Co, Tokyo, Japan) and water *ad libitum*. Systolic blood pressure was monitored in conscious mice by the tail–cuff method (MK-1030, Muromachi, Tokyo, Japan), as described previously.^39^ Angiotensin receptor blocker (ARB), telmisartan (provided by Boehringer Ingelheim, Germany) was administrated in drinking water for 2 weeks at the concentration 1 mg/kg/day. Telmisartan at this dose did not affect systolic blood pressure (data not shown). Ang-(1–7) (Peptide Institute, Osaka, Japan) was administered intraperitoneally using osmotic minipump (Model 1004 Alzet, Cupertino, CA, USA) at the concentration of 0.5 mg/kg/day for 2 weeks. Ang-(1–7) at this dose did not affect systolic blood pressure (data not shown). Antioxidant, tempol (4-Hydroxy-TEMPO; 100 mg/kg; Sigma-Aldrich, St Louis, MO, USA), was injected intraperitoneally once a day for 2 weeks.

### Morris water maze test

Spatial learning as cognitive function was evaluated by the Morris water maze test as described previously.^40,41^ In brief, each mouse was trained five times a day at 20-min intervals for 5 consecutive days. The test was performed blindly. In each trial, mice were given 120 s to find the platform. Swimming was video-tracked (AnyMaze, Wood Dale, IL, USA), and latency, path length, swim speed, and cumulative distance from the platform were recorded. The mean swim latency for all of the trials on each day in each group was calculated.

### Y maze test

The three arms were 400 mm in length and 150 mm in height, and were separated by angles of 120° (Brain Science Idea, Osaka, Japan). Each mouse was placed in the center of the apparatus and allowed to explore the maze for 8 min. Arm choices were manually recorded. Any three consecutive choices of three different arms were counted as a correct choice. The ratio of correct choice was determined by “number of alternations” divided by “total number of arm visits” minus 2.^42^

### Cerebral blood flow

After the Morris water maze test, CBF was determined using laser speckle flowmetry (Omegazone, laser speckle blood flow imager, Omegawave, Tokyo, Japan) as descried previously.^43^ Mice were anesthetized with nembutal in saline, and a midline incision was made in the scalp. Anesthesia did not significantly modify blood pressure. The skull was exposed and wet with saline. A 780-nm laser semiconductor laser illuminated the whole skull surface. The mean CBF was measured on the skull surface. Light intensity was accumulated in a charge-coupled device camera and transferred to a computer for analysis. Image pixels were analyzed to produce average perfusion values.

### Histological analysis

Mice were anesthetized followed by cardiac perfusion with ice-cold phosphate-buffered saline and 10% formalin, and their brains were immersion-fixed in 10% buffered formalin. For histological analysis, 4–5-μm-thick paraffin sections were cut, deparaffinized, and stained with hematoxylin–eosin. Samples were examined with an All-in-one fluorescence microscope (All-in-one Fluorescence Microscope BZ 9000, Keyence, Osaka, Japan) equipped with a computer-based imaging system. Cell number was determined using a computer-imaging software (Densitograph, ATTO, Tokyo, Japan).

### Real-time reverse transcriptase polymerase chain reaction method

After the Morris water maze test, the mice were overanesthetized, and the brain was removed after cardiac perfusion with ice-cold saline. The cortex and hippocampus were frozen in liquid nitrogen and stored at −80 °C until use. Total RNA was extracted from the brain with Sepazol reagent (Nacalai Tesque, Kyoto, Japan). The RNA was subjected to phenol–chloroform extraction and ethanol precipitation. Real-time quantitative reverse-transcription PCR was performed using premix Ex Taq (Takara Bio, Shiga, Japan). The level of target gene expression was normalized against glyceraldehyde-3-phosphate dehydrogenase expression in each sample. The PCR primers are shown in Supplementary Table 2.

### Detection of superoxide anion in brain sections

Detection of superoxide anion was carried out as described previously.^44^ In brief, frozen, enzymatically intact, 10-μm-thick sections were prepared from mouse brain and immediately incubated in dihydroethidium in phosphate-buffered saline for 30 min at 37 °C in a humidified chamber protected from light. Dihydroethidium (Sigma-Aldrich) is oxidized on reaction with superoxide to ethidium, which binds to DNA in the nucleus and fluoresces red. Samples were examined with an All-in-one fluorescence microscope (All-in-one fluorescence microscope BZ 9000, Keyence) equipped with a computer-based imaging system. Fluorescence of ethidium was detected with a 500- to 550-nm long-pass filter (All-in-one fluorescence microscope BZ 9000) under ×400 magnification. The value in each mouse was determined from the average of eight areas of the hippocampus. The intensity of fluorescence was analyzed and quantified using the computer-imaging software (Densitograph, ATTO).

### Western blotting

Total protein was prepared from the hippocampus of WT mice and ACE2KO mice, and western blotting was performed as previously described.^45^ Briefly, the lysate was subjected to 10% SDS-polyacrylamide gel electrophoresis, and the separated proteins were electrophoretically transferred on nitrocellulose membrane (Hybond ECL; Amersham Biosciences, Piscataway, NJ, USA). Membranes were incubated with the following specific antibodies: BDNF (1:1,000; Abcam, Cambridge, UK) and SOD3 (1:500; Santa Cruz Biotechnology, Santa Cruz, CA, USA). The bands were visualized with an enhanced chemiluminescence system (Amersham Biosciences UK, Little Chalfont, Buckinghamshire, UK). Densitometric analysis was performed using the NIH Image software (Image J, Bethesda, MD, USA).

### Statistical analysis

Values are expressed as mean±s.e.m. in the text and figures. Data were analyzed using two-way analysis of variance, followed by *post hoc* (Bonferoni method or Student’s *t*-test) analysis for multiple comparisons. Differences with *P*<0.05 were considered statistically significant.
