# Supplementary material for: Deficiency of angiotensin-converting enzyme 2 causes deterioration of cognitive function
Source: NPJ Aging Mech Dis. 2016 Oct 20;2:16024–. doi: 10.1038/npjamd.2016.24 (PMC5515001; doi:10.1038/npjamd.2016.24)
Supplement: Supplementary Information [file npjamd201624-s1.doc]

**Supplementary Information**

**Deficiency of Angiotensin Converting Enzyme 2 Causes Deterioration of Cognitive Function**

Xiao-Li Wang^1^, Jun Iwanami^1^, Li-Juan Min^1^, Kana Tsukuda^1^, Hirotomo Nakaoka^1^, Hui-Yu Bai^1^, Bao-Shuai Shan^1^, Harumi Kan-no^1^, Masayoshi Kukida^1, 2^, Toshiyuki Chisaka^1, 3^, Toshifumi Yamauchi^1, 3^, Akinori Higaki^1, 2^, Masaki Mogi^1^, and Masatsugu Horiuchi^1^*

Department of Molecular Cardiovascular Biology and Pharmacology

Ehime University, Graduate School of Medicine, Shitsukawa,

Tohon, Ehime 791-0295, JAPAN

**Supplementary Table 1: Effect of ACE2 deficiency on body weight, brain weight, brain/body ratio, systolic blood pressure and heart rate.**

|  | Body weight  (g) | Brain weight  (g) | Brain/body ratio | Systolic blood pressure (mmHg) | Heart rate  (beats/min) |
| --- | --- | --- | --- | --- | --- |
| WT | 27.06±0.193 | 0.451±0.002 | 0.0167±0.0001 | 89.71±0.84 | 513.6±18.96 |
| ACE2KO | 26.59±0.774 | 0.450±0.004 | 0.0170±0.0004 | 89.14±0.55 | 651.5±37.88* |

Values are expressed as mean±SEM (n=7-10). * p<0.05 vs WT mice.

**Supplementary Table 2**: PCR primers for quantitative RT-PCR assay

| Target | Forward (5`-3`) | Reverse (5`-3`) |
| --- | --- | --- |
| AT1R | AGTCGCACTCAAGCCTGTCT | ACTGGTCCTTTGGTCGTGAG |
| AT2R | CACTGGCAACTAAAAAGGTGTAAGA | CGGCTGCTGGTAATGTTTCTG |
| MasR | AGGTTCCCACCGCTGTGTTC | TCTTGCCCTGGGTCACTTCA |
| p22^phox^ | TGGCTACTGCTGGACGTTTCAC | CTCCAGGAGACAGATGAGCACAC |
| p40^phox^ | TTTGAGCAGCTTCCAGACGA | GGTGAAAGGGCTGTTCTTGC |
| p47^phox^ | GTCCCTGCATCCTATCTGGA | GGGACATCTCGTCCTCTTCA |
| p67^phox^ | CAGACCCAAAACCCCAGAAA | AGGGTGAATCCGAAGCTCAA |
| gp91^phox^ | TGGGATCACAGGAATTGTCA | CTTCCAAACTCTCCGCAGTC |
| TNFα | CGAGTGACAAGCCTGTAGCC | GGTGAGGAGCACGTAGTCG |
| MCP-1 | GTGTCCCAAAGAAGCTGTAGTTTT | TCATTTGGTTCCGATCCAGGTTT |
| BDNF | AGGACAGCAAAGCCACAATGT | CCTTCATGCAACCGAAGTATG |
| GADPH | TGCGACTTCAACAGCAACTC | ATGTAGGCCATGAGGTCCA |

AT1R: angiotensin II type 1 receptor; AT2R; angiotensin II type 2 receptor; TNFα: tumor necrosis factor α; MCP-1: monocyte chemotactic protein-1**;** BDNF: brain-derived neurotrophic factor; GADPH: glyceraldehyde-3-phosphate dehydrogenase
